# Ki-67 and Tumor Size in Small Bowel Tumors: Findings from an Exploratory Immunohistochemical Analysis

**DOI:** 10.3390/life15121894

**Published:** 2025-12-11

**Authors:** Laurențiu Augustus Barbu, Liliana Cercelaru, Valeriu Șurlin, Stelian-Stefaniță Mogoantă, Tiberiu Stefăniță Țenea Cojan, Daniela Marinescu, Nicolae-Dragoș Mărgăritescu, Liviu Vasile

**Affiliations:** 1 Department of Surgery, Railway Clinical Hospital Craiova, University of Medicine and Pharmacy of Craiova, 200349 Craiova, Romania; laurentiu.barbu@umfcv.ro (L.A.B.); tiberiu.tenea@umfcv.ro (T.S.Ț.C.); 2 Department of Embryology and Anatomy, University of Medicine and Pharmacy of Craiova, 200349 Craiova, Romania; liliana.cercelaru@umfcv.ro; 3 Department of Surgery, Emergency County Hospital, University of Medicine and Pharmacy of Craiova, 200349 Craiova, Romania; vsurlin@gmail.com (V.Ș.); ssmogo@yahoo.com (S.-S.M.); dtmarinescu@yahoo.com (D.M.); vliviu777@yahoo.com (L.V.)

**Keywords:** small bowel tumors, gastrointestinal stromal tumor (GIST), Ki-67, tumor size, CD117, DOG1, CD34, immunohistochemistry

## Abstract

Background: Small bowel tumors are rare and biologically diverse, and prognostic assessment remains difficult, particularly regarding proliferative markers such as Ki-67 and tumor size. Objective: To evaluate the clinicopathological and immunohistochemical characteristics of small bowel tumors and explore factors associated with malignancy. Methods: A retrospective analysis of 61 surgically treated primary small bowel tumors (2020–2024) was performed using WHO 2019/2022 and AJCC 8th criteria. Immunohistochemistry included CD117, DOG1, CD34, SMA, and Ki-67. Results: Adenocarcinomas were most frequent (52.5%), followed by GISTs (26.2%) and NETs (9.8%). CD117 and DOG1 were expressed in 93.8% of GISTs, confirming high diagnostic specificity. The median Ki-67 index was 8%, significantly higher in non-GIST tumors (*p* = 0.004). Tumor size correlated moderately with Ki-67 (ρ = 0.42, *p* = 0.018). In this exploratory model, tumor size > 5 cm (*p* = 0.03) and Ki-67 > 10% (*p* = 0.04) were associated with malignancy. Conclusions: Tumor size and Ki-67 were associated with malignancy in this exploratory multivariable analysis, but these findings should be interpreted with caution due to limited follow-up and sample imbalance. Combined with CD117/DOG1 profiling, they enhance diagnostic accuracy and may inform diagnostic assessment; however, prognostic implications require outcome-based studies.

## 1. Introduction

Primary tumors of the small intestine are rare, accounting for approximately 3% of all gastrointestinal malignancies, yet their incidence has been steadily increasing over recent decades [1]. The four predominant histological subtypes include adenocarcinomas, neuroendocrine tumors (NETs), gastrointestinal stromal tumors (GISTs), and lymphomas, each exhibiting distinct biological behaviors and therapeutic implications [1,2]. Despite technological advances, the diagnosis of small bowel tumors remains challenging due to non-specific symptoms and the limited accessibility of the small intestine to conventional endoscopy. The use of capsule endoscopy and double-balloon enteroscopy has improved detection, although most patients are still diagnosed at advanced stages [2,3].

Adenocarcinomas and NETs represent roughly 80% of small bowel malignancies, while GISTs and lymphomas account for the remainder [2]. Adenocarcinomas arise predominantly in the duodenum and are often associated with inflammatory or hereditary conditions such as Crohn’s disease and Lynch syndrome [1]. NETs occur mainly in the distal ileum and currently represent the most frequent primary small bowel cancer, with an incidence of 0.67–1.2 per 100 000 and frequent nodal or hepatic metastases at presentation [3]. GISTs constitute about 20–30% of mesenchymal small bowel tumors and demonstrate site-dependent biological heterogeneity, with higher mitotic activity and risk profiles in distal locations [4]. Small bowel lymphomas, though uncommon, comprise up to 40% of extranodal lymphomas, most often of the follicular, diffuse large B-cell, or MALT subtypes [5].

Immunohistochemistry plays a pivotal role in the diagnosis and risk assessment of these tumors. CD117 (c-KIT) and DOG1 are highly specific for GISTs, while CD34 co-expression aids in differentiating stromal from smooth-muscle lesions [6]. The Ki-67 labeling index is crucial for grading NETs, with thresholds of <3%, 3–20%, and >20% defining G1, G2, and G3 categories, respectively [4]. Evaluation of mismatch-repair (MMR) and microsatellite instability (MSI) status also assists in identifying hereditary adenocarcinomas and immunotherapy candidates [1].

The aim of this study was not simply to confirm established immunohistochemical phenotypes, but to explore whether tumor size and the Ki-67 proliferation index correlate with malignancy status within a homogeneous cohort of surgically treated primary small bowel tumors, and to describe subtype-specific patterns in an anatomical and histological context. Despite major advances in immunohistochemical and molecular profiling, large homogeneous surgical series focused exclusively on small bowel tumors remain scarce. Most studies combine gastric and intestinal sites, limiting the interpretation of site-specific correlations [2,4]. In this context, the present work provides exploratory data that may support risk stratification and diagnostic assessment in small intestinal malignancies.

## 2. Materials and Methods

### 2.1. Study Design and Patient Selection

This retrospective, cross-sectional cohort study included 61 consecutive patients with histologically confirmed primary small bowel tumors who underwent surgical treatment between January 2020 and December 2024 at the Clinical County Hospital of Craiova, Romania. All patients underwent surgical treatment during the study period, performed either with curative or palliative intent depending on the extent of disease and intraoperative findings.

Inclusion criteria were (1) patients with primary small bowel tumors confirmed by histopathological and immunohistochemical examination, and (2) availability of complete clinical, surgical, and pathological data during the study period.

Exclusion criteria included (1) metastatic lesions originating from extraintestinal primaries; (2) recurrent tumors; (3) incomplete clinical documentation or missing paraffin blocks; and (4) inadequate tissue preservation for immunohistochemical analysis.

Clinical data were retrieved from institutional electronic records and included demographic characteristics, presenting symptoms, type of surgical procedure, tumor localization, and postoperative outcomes.

Segmental resection and termino-terminal anastomosis were considered a single surgical procedure, as the anastomosis is inherently performed following bowel resection. Accordingly, cases were classified under “segmental resection/enterectomy”, and no isolated termino-terminal anastomoses without resection occurred in this cohort.

### 2.2. Study Outcomes

The primary outcome was the association between the Ki-67 proliferation index and tumor malignancy, defined according to WHO 2019/2022 histopathological criteria. Secondary outcomes included (1) the correlation between tumor size and Ki-67, (2) immunohistochemical expression patterns of CD117, DOG1, CD34 and SMA across histological subtypes, and (3) clinicopathological features by tumor type. Tumor size was measured as the largest macroscopic diameter. Ki-67 was quantified as the percentage of positive nuclei among ≥500 counted tumor cells in hotspot areas.

### 2.3. Histopathological Evaluation

All specimens were fixed in 10% neutral-buffered formalin, embedded in paraffin, and sectioned at 4 μm. Hematoxylin–eosin (H&E) staining was performed for morphological assessment. Tumors were classified according to the 2019 and 2022 World Health Organization (WHO) criteria for digestive system tumors, and pathological staging followed the 8th edition of the American Joint Committee on Cancer (AJCC) TNM classification. All NETs were classified as malignant neoplasms according to ENETS and AJCC frameworks, whereas WHO grading (G1–G3) was used to capture proliferative heterogeneity. Tumor size was measured as the largest macroscopic dimension in centimeters. Mitotic index could not be systematically evaluated because mitotic count per 5 mm^2^ was not consistently recorded in archival specimens, and hotspot-based recounting was not feasible for all cases. Therefore, the mitotic index was excluded from comparative and multivariable analyses.

### 2.4. Immunohistochemical Analysis

Immunohistochemistry was performed on formalin-fixed, paraffin-embedded (FFPE) tissue sections using an automated staining platform (Ventana BenchMark ULTRA, Roche Diagnostics, Basel, Switzerland). The following primary antibodies were used: CD117 (c-KIT, clone YR145, Dako, Glostrup, Denmark), DOG1 (clone SP31, Thermo Fisher, Waltham, MA, USA), CD34 (clone QBEnd/10, Leica, Wetzlar, Germany), SMA (smooth muscle actin, clone 1A4, Dako), and Ki-67 (clone MIB-1, Dako). Antigen retrieval was achieved using citrate buffer (pH 6.0) or EDTA (pH 8.0), depending on the antibody specifications. The EnVision detection system (Dako) with diaminobenzidine (DAB) was used for chromogenic visualization.

Staining was independently evaluated by two experienced pathologists blinded to the clinical data. Tumors were considered positive for CD117, DOG1, CD34, or SMA when at least 10% of neoplastic cells exhibited cytoplasmic or membranous staining. The Ki-67 index was calculated as the percentage of positively stained nuclei among at least 500 tumor cells in areas of highest labeling (“hot spots”). For neuroendocrine tumors, Ki-67 grading followed WHO 2019 thresholds: G1 (<3%), G2 (3–20%), and G3 (>20%). Neuroendocrine tumors were confirmed by positivity for synaptophysin and chromogranin A, although these markers were not included in the comparative immunohistochemical analysis.

### 2.5. Statistical Analysis

Data were initially processed using Microsoft Excel (Microsoft Corp., Redmond, WA, USA) with XLSTAT (Addinsoft SARL, Paris, France). Statistical analyses were subsequently performed using SPSS version 25.0 (IBM Corp., Armonk, NY, USA). Continuous variables were expressed as mean ± standard deviation (SD) or median and interquartile range (IQR), depending on data distribution assessed by the Shapiro–Wilk test. Categorical variables were summarized as frequencies and percentages.

Comparisons between groups were made using the χ^2^ test or Fisher’s exact test for categorical variables and Student’s t-test or Mann–Whitney U test for continuous variables. Correlations between continuous parameters were evaluated using Spearman’s rank correlation coefficient (ρ). Binary logistic regression was applied to explore factors associated with malignancy, and multivariate analysis of variance (MANOVA) was used as an exploratory method to evaluate combined associations among clinicopathological variables. Malignancy was defined according to WHO 2019/2022 criteria as any histologically confirmed adenocarcinoma, GIST, neuroendocrine tumor, lymphoma or leiomyosarcoma. Benign tumors included leiomyoma and non-neoplastic lesions. A *p*-value < 0.05 was considered statistically significant. Survival analysis using the Kaplan–Meier method could not be performed due to incomplete follow-up data and the limited number of recorded outcome events.

The multivariate model was designed as an exploratory analysis due to the limited sample size. Variables were selected a priori based on two criteria: (1) clinical relevance described in the literature (tumor size, Ki-67 index, CD117/DOG1 expression, age, and sex), and (2) univariate association with malignancy using a liberal significance threshold of *p* < 0.10. No automated stepwise selection procedures were applied to avoid overfitting. Therefore, the results of the multivariate analysis should be interpreted as hypothesis-generating rather than confirmatory.

### 2.6. Ethical Considerations

The study protocol was approved by the Ethics Committee of the Clinical County Hospital of Craiova (Approval No. 55515/18 November 2025). All procedures were performed in accordance with the Declaration of Helsinki. Patient anonymity and data confidentiality were strictly maintained throughout the study.

## 3. Results

### 3.1. Baseline Clinical Characteristics

The baseline characteristics of the 61 patients included in the study are summarized in Table 1. The mean age was 63.1 ± 10.2 years, with a slight female predominance (60.7%). Abdominal pain was the most common presenting symptom (67.2%), followed by anemia (31.1%) and intestinal obstruction (23.0%). Segmental enterectomy with termino-terminal anastomosis was the most frequent surgical procedure (57.4%), while entero-enteric bypass was performed in 23.0% of patients, mainly for palliative purposes. Adenocarcinoma was the most frequent histological type (52.5%), followed by GIST (26.2%) and NET (9.8%) (Table 1).

### 3.2. Histopathological and Immunohistochemical Findings

Adenocarcinoma was the most frequent histopathological subtype (52.5%), followed by GIST (26.2%) and NET (9.8%). Immunohistochemistry showed high expression of CD117 and DOG1 (93.8% each) and CD34 (68.8%) in GIST, while NETs were consistently positive for synaptophysin (100%) and frequently for chromogranin A and CD56 (83.3%). The median Ki-67 index was 8% (IQR 5–15). Most tumors presented at an advanced stage (78.7% pT2–pT4), with limited nodal (8.2%) and distant metastatic involvement (3.3%).

The anatomical distribution of tumors and the corresponding variation in tumor size and Ki-67 across histological types are summarized in Table 2. Ileal tumors were the most frequent, followed by jejunal and duodenal lesions. Adenocarcinomas were predominantly located in the duodenum, whereas GISTs and NETs occurred mainly in the jejunum and ileum, respectively. Both tumor size and Ki-67 showed distinct site- and subtype-related patterns, reflecting the heterogeneous biological behavior of small-bowel tumors.

### 3.3. Surgical Outcomes and Comparative Clinicopathological Analysis

As shown in Table 3, no significant clinical differences were observed among tumor types (*p* > 0.05). Most lesions were locally advanced (pT3–pT4), with lymph node involvement mainly in adenocarcinomas. NETs and GISTs rarely showed nodal or distant spread. Ki-67 levels were highest in adenocarcinomas and lowest in NETs, reflecting distinct proliferative patterns across groups.

Immunohistochemical analysis (Table 4) showed high CD117 and DOG1 expression in GISTs, with no positivity in other tumor types (*p* < 0.001). CD34 was also more frequently expressed in GISTs (*p* < 0.01), while SMA showed no significant differences between groups. Ki-67 levels were highest in adenocarcinomas and lowest in NETs (*p* = 0.002), reflecting distinct proliferative activity across tumor types. Among NETs (*n* = 6), Ki-67–based grading identified 3 G1 lesions (<3%) and 3 G2 lesions (3–5%), with no G3 tumors observed. NETs were therefore included in the malignant group in the exploratory model, while grading information was reported separately.

As shown in Table 5, R0 resection rates were similar across groups, with slightly lower values in adenocarcinomas. R1 margins and postoperative complications were also more frequent in adenocarcinomas compared with other tumors. Ki-67 levels remained lowest in GISTs and highest in non-GIST tumors, indicating differences in proliferative behavior among histological subtypes.

On univariate analysis, CD117, DOG1, and CD34 positivity were significantly associated with GIST histology (*p* < 0.001 for all), while no significant differences were found regarding sex distribution, SMA expression, or nodal/metastatic involvement (Table 6).

Comparative analysis of continuous variables showed no significant differences in mean age or tumor size between GIST and non-GIST tumors (Table 7). However, the Ki-67 proliferation index remained significantly higher in non-GIST tumors compared with GISTs (median 14% [8–25] vs. 6% [4–12]; *p* = 0.004). Tumor size also differed across individual tumor types in the multigroup comparison, and a moderate positive correlation was observed between tumor size and Ki-67 (ρ = 0.42, *p* = 0.018), indicating that larger tumors tended to have higher proliferative activity.

### 3.4. Correlation Analysis and Factors Associated with Proliferation

Spearman’s correlation showed a moderate positive relationship between tumor size and Ki-67 index (ρ = 0.42, *p* = 0.018), indicating that larger tumors tended to have higher proliferative activity. CD117 and DOG1 demonstrated a strong association with GIST histology (Cramer’s V = 0.82 and 0.78, respectively; both *p* < 0.001), confirming their diagnostic specificity (Table 8).

Multivariate and correlation analyses showed that tumor size >5 cm and Ki-67 >10% were associated with malignancy in this exploratory model (*p* = 0.03 and *p* = 0.04, respectively) (Table 9). In this exploratory model, Ki-67 >10% and tumor size >5 cm were associated with a histological diagnosis of malignancy (WHO criteria), rather than clinical outcomes. CD117 and DOG1 were not significantly associated with malignancy; inverse OR values reflect statistical artifact due to collinearity and sample imbalance. MANOVA showed significant overall differences between tumor types when age, size, and Ki-67 were analyzed together (Wilks’ λ = 0.71, *p* = 0.046). Cluster analysis of immunohistochemical markers identified three major subgroups, corresponding to classical GIST, partial GIST/SMA+, and non-GIST tumors.

## 4. Discussion

### 4.1. Comparison with Published Data

The incidence of small bowel tumors in our cohort aligns with global epidemiological data, confirming their rarity among gastrointestinal malignancies. Small bowel tumors represent less than 5% of all gastrointestinal neoplasms, with an estimated incidence of approximately 2–3 cases per 100,000 population per year [1,2]. Duodenal involvement is the most frequent site (50–60%), followed by jejunal and ileal locations [2,3]. The apparent increase in incidence reported over recent decades is largely attributable to advances in diagnostic imaging and endoscopic techniques, rather than a true rise in disease burden [7,8].

The histologic profile in our series, in which adenocarcinomas represented the most frequent subtype (52.5%), followed by GISTs (26.2%) and NETs (9.8%), reflects the spectrum reported in large international registries such as SEER and NADEGE, where adenocarcinomas and neuroendocrine tumors together comprise the majority of small bowel malignancies, while GISTs and lymphomas account for the remainder [8,9,10]. The predominance of duodenal adenocarcinomas and ileal NETs described in population-based cohorts supports site-specific molecular and etiologic mechanisms [3,11]. Small bowel lymphomas remain uncommon but constitute up to 30–40% of extranodal lymphomas, most frequently of follicular or diffuse large B-cell subtype [5].

Immunohistochemical findings in our cohort parallel those reported globally. GISTs demonstrated strong CD117 (c-KIT) and DOG1 expression in more than 90% of cases, with CD34 positivity in approximately two-thirds of tumors, confirming their diagnostic reliability and stromal differentiation [6,12,13,14,15]. These rates are comparable to those reported by Varshney et al. and other large multicenter series [13,14,15]. In contrast, NETs displayed a typical neuroendocrine phenotype, with universal synaptophysin expression and frequent chromogranin A and CD56 positivity, in line with current WHO criteria and contemporary literature [3,16,17,18].

For small bowel adenocarcinomas, previous studies have reported mismatch repair deficiency and microsatellite instability (MSI) in approximately 15–20% of cases [19,20]. Although MMR/MSI status was not systematically assessed in our cohort, these data underscore the need for routine MMR testing in this subtype, given its relevance for identifying hereditary cancer syndromes and for selecting patients who may benefit from immunotherapy [19,20]. For NETs, the Ki-67–based WHO classification (G1 < 3%, G2 3–20%, G3 > 20%) remains the principal prognostic determinant [16,17]. In our series, NETs showed the lowest proliferative activity among all tumor types (median Ki-67 3%, IQR 2–5), with a predominance of G1–G2 lesions, in agreement with previous reports and consistent with their generally indolent behavior [3,16,18].

Overall, our findings are consistent with current international evidence. Adenocarcinomas, GISTs, and NETs reproduced the expected clinicopathological and immunohistochemical profiles, while Ki-67 and tumor size while Ki-67 and tumor size were associated with features of higher histological grade and malignancy in this exploratory cohort. The moderate positive correlation between tumor size and Ki-67, together with their association with histological malignancy in this exploratory model, suggests that proliferative and morphological parameters may provide complementary information in the diagnostic evaluation of small bowel tumors.

In this study, malignancy reflected histological classification according to WHO criteria rather than clinical outcomes, as systematic follow-up data were not available. Therefore, the results should be interpreted as exploratory and hypothesis-generating rather than prognostic. Given the rarity of small bowel tumors, exploratory single-center data provide complementary site-specific evidence and may support hypothesis generation for future multicenter studies.

These consistent patterns support the external validity of our findings and highlight the diagnostic value of integrating CD117, DOG1, CD34, and Ki-67 in the assessment of small bowel tumors. The present study provides original data from a surgically treated cohort in an underreported population, underscoring the relevance of comprehensive histopathological and immunohistochemical characterization for improving diagnostic accuracy and supporting diagnostic work-up.

### 4.2. Immunohistochemical Profile of Small Bowel Mesenchymal Tumors

The diagnosis of small bowel mesenchymal tumors remains challenging because of overlapping spindle-cell morphology among gastrointestinal stromal tumors (GISTs), leiomyomas, and schwannomas [6,13,21]. Immunohistochemistry is therefore essential for accurate classification. Among the available markers, CD117 (c-KIT) and DOG1 (Discovered On GIST-1) are the most reliable and specific identifiers of GIST, and their combined use ensures near-complete diagnostic accuracy [2,22,23]. In our cohort, this canonical immunophenotype was confirmed, with CD117 and DOG1 positivity in 93.8% of GISTs and CD34 expression in 68.8% of cases, closely mirroring rates reported in large multicenter series [6,12,13,14,15].

**CD117**, a transmembrane tyrosine kinase encoded by the *KIT* proto-oncogene, is expressed in more than 95% of GISTs and is associated with constitutive activation of proliferative signaling through the MAPK and PI3K pathways [6]. In contrast, leiomyomas and leiomyosarcomas typically lack CD117 and DOG1 expression, but are positive for smooth-muscle markers such as SMA and desmin, while schwannomas exhibit strong S100 expression with negativity for both markers [5,13]. **DOG1**, encoded by *ANO1* on chromosome 11q13, is a calcium-dependent chloride channel expressed in interstitial cells of Cajal, the presumed progenitor cells of GIST [22]. Unlike CD117, DOG1 expression is largely independent of *KIT* or *PDGFRA* mutational status and remains detectable in CD117-negative tumors, including those harboring *PDGFRA* exon 18 mutations [9,13]. This feature makes DOG1 particularly valuable for diagnosing KIT-negative intestinal GISTs, especially in cases with atypical morphology or extensive necrosis that complicate histopathological interpretation [7].

Recent data highlight biological heterogeneity among small intestinal GISTs. Wang et al. reported increasing tumor size, mitotic activity, and CD117/DOG1 staining intensity from the duodenum to the ileum, suggesting a gradient of aggressiveness along the intestinal axis [4]. In line with these observations, most GISTs in our series presented at an advanced local stage (predominantly pT3–pT4), and their Ki-67 index, although lower than in non-GIST tumors, increased with tumor size, contributing to the overall positive correlation between size and proliferative activity (ρ = 0.42, *p* = 0.018).

Among the NETs in our cohort, Ki-67–based grading demonstrated an exclusively low-grade profile: three tumors were classified as G1 (<3%) and three as G2 (3–5%), with no G3 lesions identified. This distribution aligns with the typically indolent biological behavior of small-bowel NETs and corresponds to the lowest proliferation indices observed in ileal tumors.

Beyond their diagnostic value, CD117 and DOG1 also have prognostic and therapeutic implications, as their expression correlates with KIT or PDGFRA mutations predictive of response to tyrosine kinase inhibitors such as imatinib [1,19]. Co-expression of CD117 and DOG1 in duodenal and jejunal GISTs—often associated with larger size and higher mitotic rate—identifies lesions at increased risk of recurrence and guides adjuvant therapy decisions [4,24]. In a multicentric analysis, Varshney et al. reported CD117 and DOG1 positivity in 98% and 94% of cases, respectively, while CD34 expression (≈72%) correlated with intermediate-to-high-risk categories [13]. Similar findings were described by Terada, who reported concurrent CD117 and CD34 positivity in intestinal GISTs with intermediate mitotic indices [22]. Our rates of CD117, DOG1, and CD34 expression thus reinforce the external validity of these patterns in a surgically treated cohort.

Additional markers involved in apoptosis and cell-cycle regulation, such as p21^WAF1^ and Bax, have been associated with malignant potential, suggesting that CD117 and DOG1 expression may coexist with other molecular features of aggressive behavior [6]. The absence of CD117 and DOG1 expression in non-stromal small bowel neoplasms further enhances diagnostic specificity; duodenal lymphomas, typically CD20- or CD3-positive, consistently lack both markers, ensuring distinction from stromal tumors [5]. Accurate immunohistochemical characterization therefore enables limited resections for duodenal GISTs instead of radical pancreaticoduodenectomy, reducing morbidity without compromising oncologic outcomes [25].

According to the 2019 and 2022 World Health Organization (WHO) classifications, CD117 and DOG1 are mandatory diagnostic criteria for GISTs and should be included in all immunohistochemical panels for gastrointestinal mesenchymal tumors [3]. In our study, the integration of the Ki-67 proliferation index with CD117, DOG1, and CD34 expression provided additional insight into tumor grade and biological behavior, suggesting that Ki-67 and tumor size may act as complementary markers associated with histological malignancy in small bowel mesenchymal tumors [3,26].

### 4.3. Findings and Comparative Analysis

In our surgical cohort, adenocarcinomas represented the most frequent histological subtype (52.5%), followed by GISTs (26.2%) and NETs (9.8%), a distribution broadly consistent with large registry data for small bowel malignancies [8,9,10,11]. At the immunohistochemical level, GISTs showed the expected profile, with high CD117 and DOG1 expression (93.8% each) and CD34 positivity in approximately two-thirds of cases, in line with the diagnostic performance reported by Varshney et al. and Terada [13,22]. NETs were consistently positive for synaptophysin and frequently expressed chromogranin A and CD56, supporting their neuroendocrine lineage and the applicability of current WHO grading schemes [16,17].

As shown in Table 2, tumor location showed clear variation in both tumor size and proliferative activity. Duodenal tumors were mainly adenocarcinomas and exhibited higher Ki-67 levels, whereas jejunal lesions were predominantly GISTs and ileal lesions were enriched in well-differentiated NETs, which had the lowest proliferation indices. These site-specific patterns support the biological heterogeneity along the small intestine and help explain subtype-related differences in clinical and pathological presentation.

Statistical analyses confirmed a strong association between GIST histology and CD117/DOG1 expression (Cramer’s V = 0.82 and 0.78, respectively; both *p* < 0.001), whereas SMA showed only a weak, non-significant relationship with tumor type. The Ki-67 index was significantly lower in GISTs than in non-GIST tumors (median 6% vs. 14%; *p* = 0.004), and a moderate positive correlation between tumor size and Ki-67 (ρ = 0.42, *p* = 0.018) indicated that larger lesions tended to exhibit higher proliferative activity, echoing the size- and site-related gradients described by Wang et al. and others [4,13].

On multivariate analysis, tumor size >5 cm and Ki-67 >10% were associated with malignancy in this exploratory model (OR 3.5 and 2.9, respectively; both *p* ≤ 0.04), whereas CD117 and DOG1 did not show significant associations after adjustment for proliferation and size. These findings parallel international data showing that Ki-67 thresholds around 10–15% stratify recurrence risk and survival in both GISTs and adenocarcinomas [10,11,13,22] and that elevated Ki-67 in small bowel adenocarcinomas is linked to nodal spread and poorer prognosis [11]. In NETs, our observations are concordant with the WHO 2019/2022 framework, in which Ki-67 remains the principal grading and prognostic parameter, with higher indices associated with nodal metastasis and early recurrence [3,16,18,27,28,29,30].

Cluster analysis further underscored the immunophenotypic heterogeneity of small bowel mesenchymal tumors, delineating three main profiles: classical GIST (CD117+/DOG1+/CD34+), a partial GIST/leiomyomatous pattern (CD117+/SMA+/CD34+), and a CD117–/DOG1– non-GIST group encompassing adenocarcinomas, NETs, and lymphomas. Together, these data support an integrated approach in which tumor size and Ki-67—interpreted alongside CD117, DOG1, and CD34, and complemented by molecular testing (KIT, PDGFRA, MMR status)—may contribute to diagnostic assessment and hypothesis generation for future risk stratification [2,16,31,32,33].

### 4.4. Strengths and Limitations

This study provides a comprehensive clinicopathological and immunohistochemical characterization of surgically treated small intestinal tumors, offering valuable insight into their histologic spectrum, biomarker expression, and clinicopathological correlations. Key strengths include the inclusion of multiple histologic subtypes (GIST, adenocarcinoma, neuroendocrine tumor, lymphoma, and others) within a single, well-defined surgical cohort; the use of standardized immunohistochemical markers (CD117, DOG1, CD34, SMA, and Ki-67) for precise tumor classification; and the application of multivariable and correlation analyses that identified exploratory associations between tumor size >5 cm, Ki-67 >10%, and malignancy. Nevertheless, the study has several limitations. Its retrospective and single-center design may introduce selection bias and limit the generalizability of the findings. The relatively small sample size (*n* = 61) restricts the statistical power for subgroup comparisons, and incomplete follow-up data precluded comprehensive survival analysis. Moreover, the absence of molecular profiling (e.g., KIT, PDGFRA, or MMR status) limits deeper biological interpretation. A key limitation is the absence of systematically recorded mitotic index in GISTs, preventing standard AFIP/NCCN risk stratification. The extreme imbalance between malignant and benign cases (59 vs. 2) limits the robustness of the logistic regression model, and therefore the multivariate results should be interpreted as exploratory. Given that only two benign cases were available, the number of events was insufficient for a stable multivariable logistic regression, and including multiple predictors introduces a high risk of overfitting. Consequently, the regression output should be regarded as exploratory and hypothesis-generating rather than confirmatory. The OR values <1 observed for CD117 and DOG1 in the multivariable model do not reflect a true protective effect; rather, they result from the strong collinearity between these markers and GIST histology, combined with the extremely small number of benign cases. These coefficients are therefore statistical artifacts and should not be interpreted biologically. Because the analytic cohort included biologically distinct tumor types (adenocarcinoma, GIST, NET, lymphoma), the multivariate model captures general predictors of malignancy rather than subtype-specific determinants. The inclusion of NET G1 within the malignant category may introduce heterogeneity, as G1 tumors show indolent biological behavior, although they remain classified as malignant neoplasms in current clinical staging frameworks. Therefore, the substantial heterogeneity across tumor types limits the generalizability of these predictors, which should be interpreted as exploratory. The MANOVA results should be interpreted with caution due to the small and unbalanced group sizes; the analysis was exploratory and primarily descriptive, and nonparametric tests (e.g., Kruskal–Wallis) provide more robust comparisons across tumor types. Future prospective, multicenter studies with integrated molecular analysis are warranted to validate and expand upon these findings. Incorporating molecular correlates such as KIT, PDGFRA, and MMR status in future cohorts will not only clarify the genetic landscape of small intestinal tumors but also strengthen the translational link between immunohistochemical features and targeted therapeutic strategies. Although the cohort is small and the multivariable analysis exploratory, single-center descriptive series remain relevant in the context of rare small bowel tumors. Site-specific data are scarce, as most studies combine gastric and intestinal cases. The present dataset provides detailed histopathological and immunohistochemical characterization and may support hypothesis generation and inform the design of future multicenter studies. Accordingly, the findings should be interpreted as complementary rather than confirmatory. Comprehensive survival analysis, including Kaplan–Meier curves and Cox regression, could not be conducted because of incomplete follow-up information and the retrospective nature of the study.

### 4.5. Future Directions

Future research on small intestinal tumors should prioritize multicentric validation and the incorporation of prospective survival analyses. Given the rarity and biological heterogeneity of these neoplasms, collaborative, large-scale studies are essential to achieve representative cohorts across all histologic subtypes and to enhance the external validity of current findings.

The establishment of prospective, multicenter registries with standardized diagnostic, histopathologic, and follow-up protocols will allow more accurate identification of prognostic determinants and refinement of existing risk stratification systems. Integrating molecular and genomic profiling, as recommended by recent NCCN and ENETS guidelines, is expected to further elucidate tumor biology and identify novel therapeutic targets.

In addition, future studies should combine clinical, morphological, and molecular parameters in survival-based models to enable personalized prognostic assessment and optimize multidisciplinary treatment strategies. Such integrated approaches will contribute to the development of precision medicine paradigms for small bowel malignancies and improve patient outcomes.

In summary, the present analysis integrates clinicopathological, immunohistochemical, and proliferative parameters to provide a unified view of small intestinal tumors. The consistent association between CD117/DOG1 expression and GIST histology, together with the exploratory associations of tumor size and Ki-67 with histologic malignancy, highlights the complementary role of morphological and immunohistochemical markers in diagnostic evaluation. These results bridge diagnostic accuracy with clinical relevance, offering a preliminary descriptive framework and paving the way for molecularly integrated, multicenter validation studies.

## 5. Conclusions

In this surgically treated cohort, adenocarcinomas represented the most frequent primary small bowel tumors, followed by GISTs and NETs, consistent with contemporary epidemiological data. CD117 and DOG1 reliably confirmed GIST diagnosis with high specificity, whereas CD34 expression supported stromal differentiation. In the exploratory multivariable analysis, tumor size >5 cm and Ki-67 >10% were associated with histological malignancy; however, these observations should be interpreted with caution due to sample imbalance and the absence of systematic outcome data. A positive correlation between tumor size and Ki-67 further suggests that larger lesions may exhibit higher proliferative activity.

Overall, combining immunohistochemical markers with Ki-67 and tumor size may support diagnostic evaluation and inform future risk stratification, but does not allow definitive prognostic or therapeutic conclusions. Prospective studies with standardized follow-up and adequate statistical power are required to validate these findings.

## Figures and Tables

**Table 1:** Baseline characteristics of the study population (*n* = 61).

Parameter	Category/Description	*n* (%) or Mean ± SD
**Age (years)**	Mean ± SD (Median [IQR])	63.1 ± 10.2 (63 [56–72])
**Sex**	Female	37 (60.7%)
	Male	24 (39.3%)
**Presenting symptoms**	Abdominal pain	41 (67.2%)
	Anemia	19 (31.1%)
	Intestinal obstruction	14 (23.0%)
	Nausea/vomiting	11 (18.0%)
	Weight loss	7 (11.5%)
	Gastrointestinal bleeding (melena/hematochezia)	6 (9.8%)
	Palpable mass	4 (6.6%)
**Type of surgical intervention**	Segmental enterectomy with termino-terminal anastomosis	35 (57.4%)
	Entero-enteric bypass (palliative)	14 (23.0%)
	Limited resection/biopsy	5 (8.2%)
	Ileostomy/colostomy	4 (6.6%)
	Hemicolectomy	3 (4.9%)
**Histopathological type**	GIST	16 (26.2%)
	Adenocarcinoma	32 (52.5%)
	Neuroendocrine tumor (NET)/Carcinoid	6 (9.8%)
	Lymphoma	3 (4.9%)
	Leiomyosarcoma/Leiomyoma	2 (3.3%)
	Benign/Other	2 (3.3%)
**Ki-67 proliferation index**	Median [IQR]	8% [5–15] (range 1–40%)
**Pathologic stage**	pT2–pT4 (predominantly pT3–T4)	48 (78.7%)
	pN1 (lymph node involvement)	5 (8.2%)
	pM1 (distant metastases)	2 (3.3%)
**Tumor type**	**Marker**	***n*** **(%)**
**GIST (*n* = 16)**	CD117 positive	15 (93.8%)
	DOG1 positive	15 (93.8%)
	CD34 positive	11 (68.8%)
	SMA positive	4 (25.0%)
**Neuroendocrine tumors (NET) (*n* = 6)**	Synaptophysin positive	6 (100%)
	Chromogranin A positive	5 (83.3%)
	CD56 positive	5 (83.3%)

**Table 2:** Anatomical distribution of small bowel tumors and corresponding tumor size and Ki-67 across pathological types (*n* = 61).

Location	*n*	%	Histological Type Distribution	Median Tumor Size (cm)	Median Ki-67 (%)
**Duodenum**	9	14.8	Predominantly adenocarcinoma	5.9	14 [8–25]
**Duodenum + pancreatic extension**	1	1.6	Adenocarcinoma	—	—
**Jejunum**	15	24.6	Mainly GIST + NET	6.8 (GIST subset)	6 [4–12] (GIST)
**Ileum**	35	57.4	NET + GIST predominant	3.2 (NET subset)	3 [2–5] (NET)
**Jejunum + ileum**	1	1.6	GIST	6.8 (GIST subset)	6 [4–12] (GIST)
**Total**	61	100	Adenocarcinoma 32; GIST 16; NET 6; Lymphoma 3; Other 4	—	Overall: 8 [5–15]

**Table 3:** Clinical, Surgical, and Pathological Overview of the Study Population (*n* = 61).

Variable	GIST (*n* = 16)	Adenocarcinoma (*n* = 32)	NET (*n* = 6)	Others (*n* = 7)	*p*-Value
**Mean age (years)**	61.8 ± 9.7	66.2 ± 8.9	59.5 ± 11.2	64.0 ± 10.1	0.22
**Female sex (%)**	58.3%	66.7%	50.0%	57.1%	0.64
**Abdominal pain (%)**	72.2%	50.0%	66.7%	57.1%	0.38
**Intestinal obstruction (%)**	19.4%	41.7%	16.7%	28.6%	0.33
**Segmental resection (%)**	63.9%	50.0%	50.0%	57.1%	0.61
**Pathologic stage pT2 (%)**	11.1%	8.3%	33.3%	14.3%	—
**Pathologic stage pT3 (%)**	44.4%	41.7%	50.0%	28.6%	—
**Pathologic stage pT4 (%)**	33.3%	50.0%	16.7%	57.1%	—
**Lymph node involvement (pN1, %)**	2.8%	33.3%	0%	14.3%	—
**Distant metastasis (pM1, %)**	5.6%	8.3%	0%	0%	—
**Mean Ki-67 index (%)**	8.7	11.2	4.5	7.3	—
**Tumor size (cm) median [IQR]**	6.8 [4–9.5]	5.9 [3.2–8.0]	3.2 [2–5]	4.5 [3–6]	0.035

**Table 4:** Immunohistochemical Expression by Tumor Type.

Marker	GIST (%)	Adenocarcinoma (%)	NET (%)	Others (%)	*p*-Value
**CD117**	93.8	0.0	0.0	0.0	<0.001
**DOG1**	93.8	0.0	0.0	0.0	<0.001
**CD34**	68.8	0.0–20.0	0.0	0.0–28.6	<0.01
**SMA**	25.0	~15.0	33.3	~28.6	0.52
**Ki-67 (%) median [IQR]**	8 [5–15]	18 [10–25]	3 [2–5]	8 [5–10]	0.002

**Table 5:** Surgical Outcomes and Clinicopathological Correlations.

Parameter/Variable	GIST (*n* = 16)	Adenocarcinoma (*n* = 32)	NET (*n* = 6)	Others (*n* = 7)	Non-GIST Total (*n* = 45)	*p*-Value
**Complete resection (R0)**	13 (80.6%)	21 (66.7%)	5 (83.3%)	6 (85.7%)	32 (71.1%)	—
**R1 margin involvement**	2 (11.1%)	8 (25.0%)	0	1 (14.3%)	9 (20.0%)	—
**Postoperative complications**	3 (19.4%)	11 (33.3%)	1 (16.7%)	1 (14.3%)	13 (28.9%)	—
**Mean age (years)**	61.8	—	—	—	65.4	0.18
**Female sex (%)**	58.3%	—	—	—	63.0%	0.64
**Tumor size (cm, mean)**	6.8	—	—	—	5.3	0.11
**Ki-67 median (%)**	6	—	—	—	12	0.01

**Table 6:** Comparative analysis of categorical variables between GIST and non-GIST tumors (*n* = 61).

Variable	Statistical Test	χ^2^/Fisher Value	*p*-Value	Interpretation
**CD117 positivity**	Chi-square test	χ^2^ = 42.3	**<0.001**	**Significant** (GIST strongly associated with CD117+)
**DOG1 positivity**	Chi-square test	χ^2^ = 38.6	**<0.001**	**Significant** (DOG1 highly specific for GIST)
**CD34 positivity**	Chi-square test	χ^2^ = 12.7	**0.001**	**Significant** (frequent in GIST)
**SMA positivity**	Fisher’s Exact test	—	**0.42**	Not significant
**Sex (female/male)**	Chi-square test	χ^2^ = 0.28	**0.60**	Not significant
**pN1 (yes/no)**	Fisher’s Exact test	—	**0.09**	Not significant trend (adenocarcinomas more likely pN1)
**pM1 (yes/no)**	Fisher’s Exact test	—	**0.47**	Not significant
**Stage pT (T2–T4)**	Chi-square test for trend	χ^2^ = 1.92	**0.38**	Not significant (no clear trend by tumor type)

**Table 7:** Comparative analysis of continuous variables by tumor type.

Variable	Groups Compared	Statistical Test	Mean/Median Values	Test Value	*p*-Value	Interpretation
**Age (years)**	GIST (*n* = 16) vs. non-GIST (*n* = 45)	Independent t-test	61.8 ± 9.7 vs. 65.4 ± 8.9	t = 1.42	0.16	NS (similar age distribution)
**Tumor size (cm)**	GIST vs. non-GIST	Mann–Whitney U	6.8 [4.0–9.5] vs. 5.3 [3.2–7.0]	U = 324.5	0.21	NS (no size difference)
**Ki-67 (%)**	GIST vs. non-GIST	Mann–Whitney U	6 [4–12] vs. 14 [8–25]	U = 102.5	0.004	Significant (higher proliferation in non-GIST)
**Tumor size (cm)**	GIST (*n* = 16), Adenocarcinoma (*n* = 32), NET (*n* = 6), Lymphoma (*n* = 3)	Kruskal–Wallis	medians: 6.8/5.9/3.2/4.5	H = 6.72	0.035	Significant differences across groups
**Ki-67 (%) vs. tumor size**	All tumors (*n* = 61)	Spearman correlation	ρ = 0.42	Z = 2.43	0.018	Positive correlation (larger tumors → higher Ki-67)

**Table 8:** Correlation analysis between clinicopathological and immunohistochemical parameters.

Relationship	Statistical Test	Correlation Coefficient	*p*-Value	Interpretation
**Tumor size ↔ Ki-67 index**	Spearman’s ρ	**0.42**	**0.018**	*Moderate positive correlation* (larger tumors show higher proliferation)
**Age ↔ Tumor size**	Spearman’s ρ	0.12	**0.34**	*No significant correlation*
**Age ↔ Ki-67 index**	Spearman’s ρ	–0.09	**0.48**	*No significant association between age and proliferation*
**CD117 ↔ Tumor type**	Cramer’s V (post χ^2^)	**0.82**	**<0.001**	*Strong association* (CD117 highly specific for GIST)
**DOG1 ↔ Tumor type**	Cramer’s V	**0.78**	**<0.001**	*Strong association* (DOG1 strongly related to GIST)
**CD34 ↔ Tumor type**	Cramer’s V	0.46	**0.008**	*Moderate association*
**SMA ↔ Tumor type**	Cramer’s V	0.21	**0.29**	*Weak, non-significant association*

**Table 9:** Summary of multivariate and correlation analyses.

Analysis Type	Variables Tested	Statistical Method	Main Results/Coefficients	*p*-Value	Interpretation
**Binary logistic regression**	Tumor size >5 cm	OR = 3.5 [1.1–11.1]	**Significant predictor of malignancy**	**0.030**	Larger tumors associated with malignancy
	Ki-67 > 10%	OR = 2.9 [1.0–8.3]	**Significant association (exploratory)**	**0.040**	Higher proliferation → malignancy
	CD117 positivity	OR = 0.4 [0.1–1.2]	Not significant; OR < 1 reflects collinearity	0.120	Not independent
	DOG1 positivity	OR = 0.5 [0.2–1.4]	Not significant; OR < 1 reflects collinearity	0.180	—
	Age (per 10 years)	OR = 1.2 [0.8–1.9]	Not significant	0.330	—
	Sex (male)	OR = 1.1 [0.4–3.3]	Not significant	0.820	—
**Multivariate analysis (MANOVA)**	Age, Size, Ki-67 vs. Tumor Type	Wilks’ λ = 0.71	Global model significant	**0.046**	Combined parameters differ by tumor type
	Post hoc: Ki-67 (Adeno > GIST)	—	**Significant**	**0.004**	Adenocarcinomas more proliferative
**Exploratory cluster analysis**	CD117, DOG1, CD34, SMA	Hierarchical (Ward/Euclidean)	3 clusters identified:	—	—
	Cluster 1	Algorithm-defined GIST-like pattern *(NOT actual CD117/DOG1 positivity)*	28 cases (45.9%)	—	Similarity-based cluster; does not reflect true IHC expression
	Cluster 2	Mixed smooth-muscle/stromal similarity profile	8 cases (13.1%)	—	Heterogeneous cluster with partial leiomyomatous features
	Cluster 3	Non-GIST-like pattern (marker-negative dominant)	25 cases (41.0%)	—	Corresponding broadly to adenocarcinoma, NET, lymphoma

Note: Cluster patterns reflect unsupervised similarity analysis and do not indicate true immunohistochemical positivity. Actual CD117 and DOG1 expression is restricted to GISTs, as shown in Table 4. OR < 1 for CD117 and DOG1 reflects collinearity with GIST histology and extreme class imbalance, not a protective effect. The model is exploratory.

## Data Availability

The data presented in this study are available on request from the corresponding author. The data are not publicly available due to patient confidentiality.
